# Computed tomographic features of severe horn infection in a male Scimitar‐horned oryx (*Oryx dammah*)

**DOI:** 10.1111/vru.13440

**Published:** 2024-10-09

**Authors:** Kayla Le, Victoria Riggs, Seng Wai Yap, Martina Ernestova, Kimberley Sebastian

**Affiliations:** ^1^ Department of Surgical Sciences School of Veterinary Medicine University of Wisconsin Madison Wisconsin USA; ^2^ Department of Pathobiological Sciences School of Veterinary Medicine University of Wisconsin Madison Wisconsin USA

**Keywords:** emphysematous osteomyelitis

## Abstract

A 3‐year‐old male Scimitar‐horned oryx presented for evaluation of an acutely deformed right horn with right head tilt and right facial pain. Computed tomographic evaluation revealed an increased volume of central fluid/soft tissue attenuation with gas‐attenuating foci within the right horn. The right horn was amputated at the right horn base. Imaging and histopathologic features were consistent with emphysematous osteomyelitis. Following treatment, the patient returned to normal behavior. This is the first veterinary report describing the computed tomographic features of a horn infection in a Scimitar‐horned oryx.

## 1 SIGNALMENT, HISTORY, AND CLINICAL FINDINGS

A 3‐year‐old male Scimitar‐horned oryx (*Oryx dammah*) privately owned by a safari park presented to the University of Wisconsin Madison Veterinary Medical Center (UWVC) for skull CT due to subacute horn deformation first noted 2 months prior to presentation. A right head tilt and right ear pressing were first noted 6 months prior to the presentation. Skull radiographs performed by the referring veterinarian were inconclusive at that time. Ear culture was negative. The patient was treated with dexamethasone, ivermectin, and ceftiofur crystalline‐free acid. Clinical signs improved following treatment but were still noted intermittently.

Approximately 2 months prior to presentation to the UWVC, the right horn began to droop dramatically and progressively. Oryx herd fecal examination revealed a high *Strongyle* spp. count, and the oryx was dewormed with ivermectin and then moxidectin. One month prior to presentation, the oryx was additionally examined for head tilt, twitching, and right facial pain. Following this examination, the oryx was treated with flunixin meglumine and enrofloxacin, which resulted in minimal improvement in clinical signs.

On presentation, the oryx was alert and responsive. A visual examination was conducted following safe work practices for handling wildlife. The oryx permitted an abbreviated manual palpation, which detected increased warmth over the base of the right horn. The right horn was pointed ventrally, beginning approximately 12 cm from its base. No nasal discharge was noted.

## 2 IMAGING, DIAGNOSIS, AND OUTCOME

**FIGURE 1:**
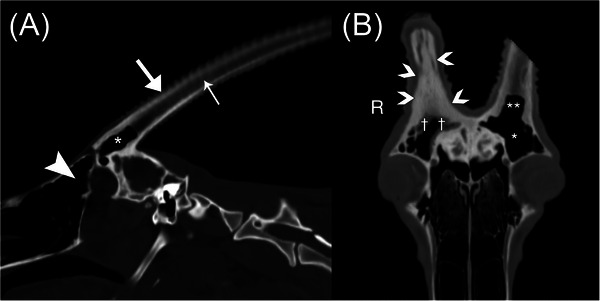
A, Multiplanar reconstructions in the sagittal plane using a bone algorithm (window width 1600; window level 500) of the normal gas‐filled left frontal sinus (arrowhead), cornuate process with gas‐filled cornual diverticula (*) and horn demonstrating normal thick mineralized outer keratin layer (wide arrow) and thinner central hypoattenuating soft tissue horn core (narrow arrow). B, Multiplanar reconstruction in a dorsal oblique plane using a bone algorithm (window width 2500; window level 500) demonstrating unilateral soft tissue to fluid attenuating lining of the right frontal sinus extending to the cornuate diverticulum (white †) with comparison to the normal air‐filled left frontal sinus (white *) and cornuate diverticulum (white **). Note the irregular cornified epithelial margin surrounding the bony horn core on the right side. Concurrent osseous proliferation fills the right cornuate diverticulum and the cornuate process (white arrowheads), and concentric smooth osseous proliferation extends into the right frontal sinus.

Noncontrast enhanced CT of the head and the cervical region was performed under general anesthesia with the oryx positioned in sternal recumbency using a multidetector 16‐slice CT scanner (GE Lightspeed, GE Healthcare) using bone and detail algorithms (parameters: pitch 0.5, 140 kVP, 225 mAs, 1.25 mm slice thickness). The distal portion of the ventrally displaced right horn was amputated using hoof nippers to accommodate the patient within the CT bore. The amputated horn section was noted to be centrally hollow. Dorsal and sagittal multiplanar reconstruction with soft tissue and bone windows were performed to assess the extent of involvement (Figure 1).

**FIGURE 2:**
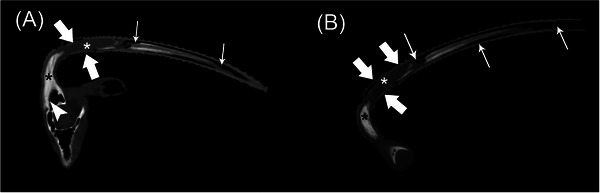
CT sagittal plane, multiplanar reconstructions using a bone algorithm (window width 1600; window level 500) of the (A) centered at the proximal aspect of the right frontal sinus, cornuate process and horn, and (B) distal aspects of the cornuate process and horn. Smooth osseous proliferation is present along the dorsal margin of the right frontal sinus and completely fills the cornual diverticula (black *). Mild soft tissue to fluid attenuating material adheres to the lumen of the frontal sinus (arrowhead, A). The outer mineralized keratin layer of the proximal horn has multifocal defects (wide arrows) and is thinned throughout the horn length. The proximal horn core is filled with reduced mineral and hypoattenuating soft tissue material (white *) with variably sized gas foci and complete gas filling of the distal horn core (narrow arrows). The right horn has an abnormal ventrolateral angle compared to the left.

**FIGURE 3:**
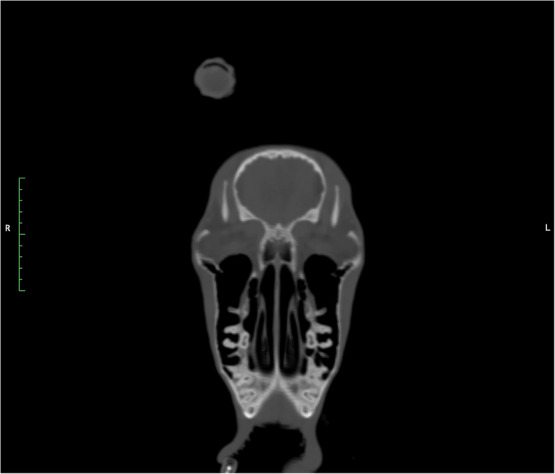
Video of a CT dorsal oblique plane, multiplanar reconstruction in a bone algorithm (window width 2834; window level 633) demonstrating the asymmetric shape of the right horn, remodeling of the right frontal sinus, cornuate process, and concurrent lysis of the outer mineralized keratin layer and horn core with adjacent gas within the right horn.

The CT study revealed right horn asymmetry characterized by malformation of the horn, which was angled caudoventrally and laterally (Figure 2). At the base of the skull, the right frontal bone and cornuate process of the right horn were circumferentially thickened with smooth periosteal proliferation, most severe along the dorsal aspects. The right cornuate process extended into the proximal aspect of the right horn core, with mineral attenuating tissue filling the cornual diverticula (Figure 3). There were multifocal defects within the proximal aspect of the outer mineral attenuating keratin layer of the right horn, with thinning of the outer keratin layer throughout the length of this horn. This caused expansion of the right horn core, which was filled with mixed thin, linear, mineral attenuating, hypoattenuating soft tissue content (Hounsfield units [HU] ranging from 30 to 70), and gas. The distal aspect of the right horn core was entirely gas‐filled. The left horn was characterized by a thicker outer keratin mineral attenuating rim, with a thinner centrally hypoattenuating horn core (HU 109 to 122).

Within the right ventral nasal cavity, middle nasal conchae, caudoventral aspect of the right maxillary sinus, and adhered to the luminal margin of the right frontal sinus, was a mild amount of soft tissue to fluid attenuating material. No evidence of nasal turbinate lysis was identified. Additionally, within the right external ear canal was a mild amount of luminal lobular soft tissue attenuating debris. Bilaterally, the tympanic bullae/cavity were normal.

Caudal to the epiglottis and caudal to the cranial esophageal sphincter, at the level of C1–C2, was a well‐defined, ovoid, faintly hyperattenuating (HU ∼50) space‐occupying structure measuring 2.25 cm *H* × 1.79 cm *W* × 3.72 cm *L*. This structure was closely associated with the retropharyngeal soft tissues and dorsal esophageal wall, coursing within the esophageal lumen and ventrolaterally surrounded by gas. Within the cervical soft tissues adjacent and caudal to the left dorsolateral margin of the previously described ovoid structure was a left deep cervical lymph node that was asymmetrically enlarged. Additionally, the left thyroid gland was asymmetrically centrally focally hypoattenuating and focally enlarged (0.98 cm H) compared with cranial and caudal poles, likely incidental to this case. The remainder of the structures were within normal limits, and facial muscles were bilaterally symmetric.

**FIGURE 4:**
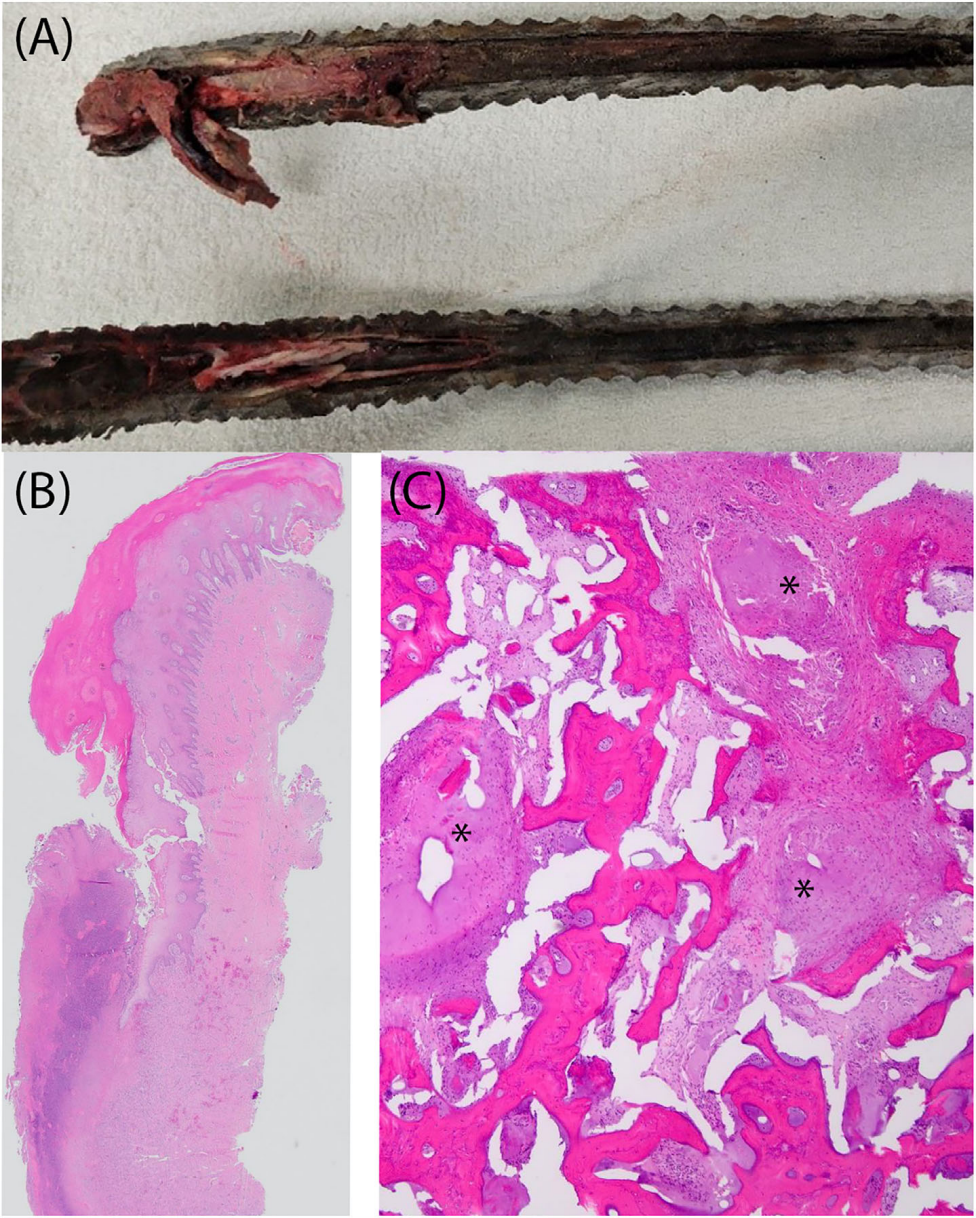
A, Gross image of partially amputated horn on sagittal plane cut section demonstrating necrotic tissues within the horn core. B, On the histopathologic section, the superficial horn is regionally extensively ulcerated and necrotic with underlying dermal granulation tissue. Normal horn is shown at the top, blending into the necrotic tissue. H&E stain. 4× objective. C, The bone is regionally replaced by necrotic debris (black *). H&E stain. 10× objective.

The right horn was amputated at the level of the haired skin; the frontal sinus cavity was not visibly entered. On the cut section, the central portion of the horn was noted to contain pale tan to red, soft, and friable tissue approximately 10 cm from the base of the horn (Figure 4). Histopathologic evaluation revealed regionally extensive horn keratin and epithelium disruption and replacement by abundant necrotic debris and many colonies of large cocci bacteria. Underlying this necrotic coagulum were numerous plump fibroblasts within collagenous stroma containing many perpendicularly oriented capillaries (granulation tissue). The underlying bone was almost completely replaced by large amounts of hemorrhage and fibrin. In better‐preserved areas of bone were peripheral moderate infiltrates of degenerate neutrophils and necrotic debris. The proximal base of the horn was within normal limits.

Aerobic and anaerobic cultures resulted in the heavy growth of a mixed bacterial population. Colonies with significant growth included *Clostridium sporogenes, Peptoniphilius* spp.*, Klebsiella oxytoca, Escherichia coli*, and *Providencia alcalifaciens*.

The imaging and histologic findings were most consistent with emphysematous osteomyelitis with associated multifocal keratin defects and abnormal horn growth with angulation. Additionally, right frontal bone hyperostosis or reactive periostitis and nondestructive right‐sided rhinitis and sinusitis were noted.

Ampillicin beads were placed in the wound bed, and the region was bandaged. The patient was treated with ceftiofur crystalline‐free acid and meloxicam. No clinical signs could be attributed to the esophageal mass, and the patient had no prior history of dysphagia. Further investigation of the esophageal nodule was declined, and the patient was discharged for further care at home. At 2 months postoperatively, the patient was reported to have returned to normal behavior with no further head tilt or ear pressing.

## 3 DISCUSSION

This case report describes CT, gross, and histologic characteristics of a severe bacterial infection in the horn of a Scimitar‐horned Oryx with secondary intraosseous pneumatosis and osteomyelitis. Intraosseous gas displaying the “pumice stone” pattern, defined as clusters of multiple foci of intramedullary gas with variable sizes and shapes, pathognomonic for emphysematous osteomyelitis, was noted in 96% of cases in one retrospective.^1^ In contrast, degenerative causes of intraosseous gas, such as pneumatocysts or subchondral cysts, have been reported to have a thin sclerotic rim along the periphery of the intraosseous gas locules. Noninfectious causes of intraosseous gas were noted to have less variably sized air foci, suggesting preservation of the intramedullary bone trabeculations. These trabeculations are rapidly destroyed in cases of emphysematous osteomyelitis, leading to variable shapes and sizes of the intramedullary gas foci. Other causes of intraosseous air include open fractures, the “intravertebral vacuum cleft sign” of a vertebral collapse or fracture, as well as malignant processes leading to osteonecrosis.^2^, ^3^, ^4^ Additional imaging findings associated with emphysematous osteomyelitis included adjacent soft tissue emphysema and a lack of associated cortical destruction.^1^

Although rarely reported, the most common sites of involvement in humans were the pelvic bones, vertebral bodies, and femurs.^1^ Emphysematous osteomyelitis may develop secondary to an intra‐abdominal infection or surgery, and diabetes mellitus and malignancy may be risk factors for its development.^5^, ^6^, ^7^ Emphysematous osteomyelitis was previously reported in the humerus of a dog who was being treated with chemotherapy.^8^ To the best of the authors’ knowledge, emphysematous osteomyelitis has not been previously reported in a large animal species.

Osteomyelitis may develop through hematogenous spread or, as is more likely in this case, from exogenous sources.^9^ Scimitar‐horned oryx are susceptible to trauma that may deform, crack, or split the horn.^10^ We speculate an external injury to the horn allowed the introduction of bacteria in this case. The resulting osteomyelitis led to a weakening of the horn and a change in horn shape. Additional differentials for the horn asymmetry may include developmental or neoplastic causes.^11^, ^12^ Imaging findings of space‐occupying gas‐filled spaces in the horn core, histopathology of osteomyelitis, and culture results positive for anaerobic gas‐producing bacteria support the diagnosis of emphysematous osteomyelitis.

In domestic cattle, bacteria introduced at the horn secondary to horn injury, dehorning, or tipping of horns may result in frontal sinusitis noted by frontal bone distortion and nasal discharge as the frontal sinus contains a cornual extension in this species.^13^, ^14^ The anatomy of the frontal sinus, as well as the degree of pneumatization of the horncore in members of the Bovidae family, is variable.^15^, ^16^ The anatomy of the Scimitar‐horned oryx sinuses has not been previously well described. The frontal sinus of the related Oryx gazelle has been described as being contained exclusively within the frontal bone with caudal extension to the frontoparietal suture and into the base of the horncore. Additionally, the frontal sinus was noted to contain numerous bony struts that subdivided the sinus.^15^ Computed tomographic examination of this patient revealed a small extension of the cornuate process of the frontal sinus into the proximal horn core with concurrent rhinitis and sinusitis.

The present report represents the first report to describe CT findings associated with a horn infection in a Scimitar‐horned oryx. Additionally, this report provides information on the sinus anatomy of the Scimitar‐horned oryx. Clinicians should be aware that deformation and the finding of several small, variably sized, and shaped intraosseous gas foci in the horn may be due to emphysematous osteomyelitis.

## LIST OF AUTHOR CONTRIBUTIONS

**Category 1**

(a) Conception and design: Le, Riggs, Yap, Ernestova, Sebastian
(b) Acquisition of data: Le, Riggs, Yap, Ernestova, Sebastian
(c) Analysis and interpretation of data: Le, Riggs, Yap, Ernestova, Sebastian

**Category 2**

(a) Drafting the article: Le, Riggs, Yap, Ernestova, Sebastian
(b) Reviewing article for intellectual content: Le, Riggs, Yap, Ernestova, Sebastian

**Category 3**

(a) Final approval of the completed article: Le, Riggs, Yap, Ernestova, Sebastian

## CONFLICT OF INTEREST STATEMENT

The authors declare no conflict of interest.

## PREVIOUS PRESENTATION OR PUBLICATION DISCLOSURE

none

## REPORTING CHECKLIST DISCLOSURE

none
